# Beyond symptom severity: Prognostic information from subjective person‐specific problem networks in young people

**DOI:** 10.1002/jcv2.70189

**Published:** 2026-11-11

**Authors:** Felix Vogel, Stefanie Jungmann

**Affiliations:** ^1^ Department of Clinical Psychology and Psychotherapy of Childhood and Adolescence Institute of Psychology Johannes Gutenberg University Mainz Mainz Germany

**Keywords:** developmental psychopathology, personalized assessment, person‐specific networks, problem breadth, prognosis, transdiagnostic psychopathology, young people

## Abstract

**Background:**

Person‐specific network models are increasingly proposed to support personalized assessment and psychotherapy, but it remains unclear whether the information they contain is prospectively informative in young people. We tested whether characteristics derived from a brief perceived causal network (PECAN), particularly its perceived relational structure, provided prognostic information about subsequent psychopathology beyond baseline symptom severity.

**Methods:**

Participants were 361 children, adolescents, and young adults assessed by self‐report or parent‐report. At baseline, participants selected personally relevant problems, rated their severity, and indicated how they perceived these problems to be related. Psychopathology was assessed with the pediatric symptom checklist (PSC‐D) at baseline and approximately 4, 8, and 12 weeks later. Linear mixed‐effects models examined prospective associations of PECAN characteristics with psychopathology. Exploratory post hoc analyses additionally examined problem breadth, defined as the number of personally relevant problems selected.

**Results:**

Global characteristics of perceived problem relations showed little consistent incremental prognostic information beyond baseline psychopathology and problem breadth. Mean perceived causal strength and maximum normalized weighted outdegree were not associated with subsequent psychopathology, while an inverse association with network density emerged only in self‐report and was not robust across sensitivity analyses. In exploratory analyses, greater problem breadth was associated with higher subsequent psychopathology in both self‐report and parent‐report, and these associations remained essentially unchanged after adjustment for conventional PSC‐D symptom breadth.

**Conclusion:**

The first prospective evaluation of PECANs provided limited support for incremental prognostic information contained in global perceived relational characteristics. Exploratory analyses nevertheless identified problem breadth as a potentially informative compositional characteristic of personalized problem representations. These findings suggest that different components of person‐specific models should be evaluated separately and highlight the need for confirmatory replication before they inform personalized clinical decisions.

## Key Points

**What's known?**

- Person‐specific network models are increasingly proposed to support personalized assessment and psychotherapy, but their prospective validity in young people is largely unknown.

**What's new?**

- In 361 children, adolescents, and young adults, global characteristics of perceived problem relations showed little consistent incremental prognostic information beyond baseline psychopathology and problem breadth. In exploratory analyses, greater problem breadth was associated with higher subsequent psychopathology in both self‐ and parent‐report, even after accounting for conventional symptom breadth.

**What's relevant?**

- The findings provide a first prospective evaluation of PECANs and suggest that brief person‐specific assessments may add clinically relevant information in child and adolescent mental health. Different components of personalized network models should be validated separately before informing clinical decisions.

## INTRODUCTION

Person‐specific models of psychopathology are increasingly proposed as a basis for personalized assessment and psychotherapy, including individualized case conceptualization and the identification of potentially relevant treatment targets (Bringmann, 2021; Burger et al., 2022; von Klipstein et al., 2020; Wright & Woods, 2020). The relevance of such personalization is also supported in young people: a recent meta‐analysis of randomized trials found a small but significant advantage of personalized over standardized psychological interventions in adolescents, while also highlighting substantial heterogeneity in how personalization was implemented (Li et al., 2024). Young people are a particularly important population in which to evaluate person‐specific approaches because many mental disorders first emerge during childhood, adolescence, and early adulthood, making this a critical period for early assessment and intervention (Solmi et al., 2022). Person‐specific representations may be especially relevant during this period because psychological difficulties can be heterogeneous and may change substantially across development. Person‐specific network approaches represent one potential route toward such personalization because they conceptualize psychopathology as a system of interacting problems while allowing the configuration of that system to be characterized at the level of the individual (Borsboom, 2017; Bringmann, 2021). Such representations may preserve clinically relevant heterogeneity that is obscured when individuals are summarized by diagnostic categories or aggregate symptom scores, as individuals with similar overall symptom severity can differ substantially in the particular problems contributing to that severity (Fried & Nesse, 2015; Sonuga‐Barke, 2016).

As person‐specific networks move toward clinical applications, establishing the validity of the information they provide becomes increasingly important. Their use for personalization rests on one fundamental empirical assumption: characteristics captured by these models should contain information relevant to subsequent clinical course. Initial evidence from statistically estimated person‐specific longitudinal networks is promising but limited. In adults receiving psychotherapy for chronic depression, person‐specific symptom‐network characteristics improved prediction of treatment outcome beyond conventional baseline characteristics, with pairwise symptom associations contributing particularly strongly (Schumacher et al., 2024). A reanalysis of the same data supported incremental information from symptom associations beyond changes in symptom severity (Schumacher et al., 2026). A smaller proof‐of‐concept study likewise found prospective associations between idiographic network characteristics and depression outcomes 6 months later (Cusack et al., 2024). Together, these findings suggest that person‐specific network information may be prospectively informative, but available evidence is based primarily on networks estimated from intensive repeated assessments.

Perceived Causal Networks (PECANs) offer a substantially lower‐burden approach to constructing person‐specific networks (Klintwall et al., 2023; Vogel, Blanken, et al., 2025). Rather than estimating relations statistically from repeated measurements across days or weeks, individuals construct a network within a single assessment by selecting psychological problems they consider personally relevant, rating their current severity, and indicating whether and how strongly they perceive these problems to influence one another (Vogel, Blanken, et al., 2025). The resulting network therefore represents the individual's subjective understanding of their difficulties rather than statistically established or objectively causal relations. At the same time, evaluating PECANs in young people raises a specific developmental question because the method requires both recognition of personally relevant difficulties and judgments about how these difficulties influence one another. These components may not be equally accessible across development. This raises the possibility that the compositional and relational components of PECANs may also differ in their validity. Previous PECAN‐CA research found that older age and better understanding of symptom and network concepts were associated with more reliable network representations and greater feasibility (Vogel, Fromm, et al., 2025).

Previous research supports several prerequisites for investigating the prospective validity of PECANs. In adults, PECANs were highly individualized and showed good immediate reliability (Klintwall et al., 2023), while the child and adolescent adaptation demonstrated good feasibility and reliability for several characteristics (Vogel, Fromm, et al., 2025). Young people with similar social‐anxiety presentations also rated their personalized PECANs as more representative than randomized or prototypical disorder representations (Vogel et al., 2026). These findings suggest that PECANs can efficiently capture person‐specific differences that respondents recognize as representative of their difficulties. Whether such differences also contain information about subsequent psychopathology, however, remains unknown.

A particularly demanding benchmark for evaluating such information is current psychopathology. Baseline symptom severity is itself strongly prognostic: in more than 11,000 young people, current symptoms predicted transition into the highest psychopathology‐risk group one year later with an area under the receiver operating characteristic curve of 0.84 (Hill et al., 2025). For a personalized representation to provide meaningful additional prognostic information, it therefore needs to capture characteristics of an individual's difficulties that are not already contained in overall symptom severity.

Importantly, PECANs contain two conceptually distinguishable layers of personalized information. First, the selection of problems characterizes the composition of an individual's difficulties, including their breadth—that is, how many different problems are considered personally relevant. Second, the perceived relations characterize how these selected problems are subjectively organized as a network (Vogel, Blanken, et al., 2025). As illustrated in Figure 1, young people with comparable levels of aggregate psychopathology may therefore construct personalized representations that differ both in the breadth and composition of selected problems and in how these problems are perceived to be connected.

**FIGURE 1:**
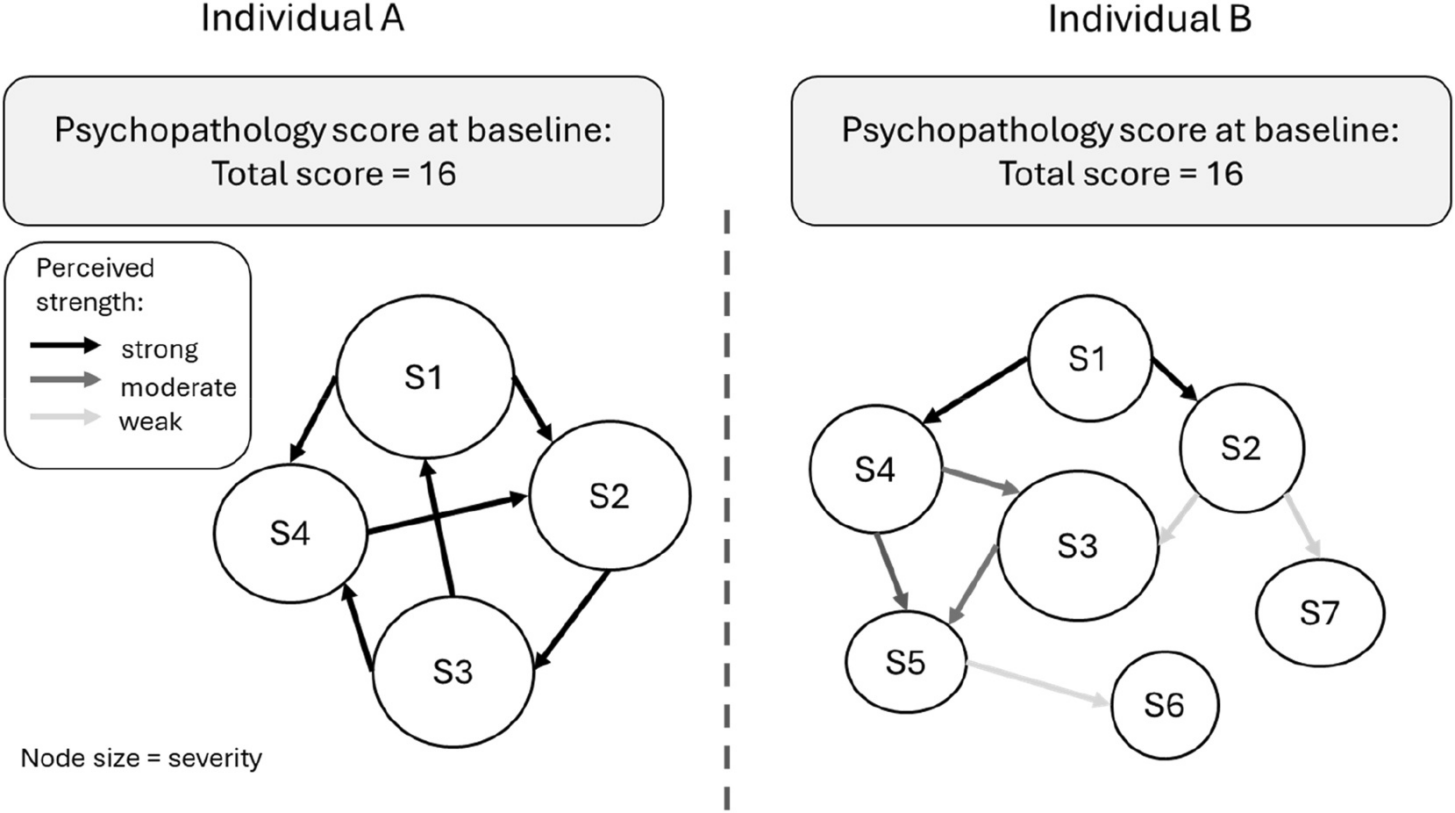
Same baseline psychopathology, different person‐specific problem representations. Schematic illustration of two individuals with the same baseline PSC‐D total score but different person‐specific configurations of psychological problems. S1–S7 denote individually selected problems and do not correspond to fixed PSC‐D items. Despite identical overall psychopathology scores, the individuals differ in which and how many problems they consider personally relevant (problem breadth), the severity of these problems (represented by node size), and how they perceive the problems to be related to one another. Arrow width and shading represent perceived strength of influence. Thus, identical conventional psychopathology scores may correspond to substantially different person‐specific configurations of problems. PSC‐D, pediatric symptom checklist.

This distinction allows a more specific question than whether a person‐specific network as a whole is prognostically informative: where within the representation does prospective information reside? Personalized problem selection may preserve heterogeneity that is lost in aggregate scores. Previous longitudinal research provides indirect evidence that higher psychiatric symptom counts in adolescence and difficulties spanning multiple problem domains are associated with poorer subsequent outcomes (Vander Stoep et al., 2002; Verhulst & van der Ende, 1993), while greater transdiagnostic symptom diversity has been associated with higher distress and lower well‐being (Kennedy et al., 2025). However, these approaches generally do not separate breadth from overall symptom burden. The relational component of a PECAN may, in turn, provide additional information about how an individual's problems are perceived to reinforce or influence one another. Whether either component carries prospective information beyond conventional symptom severity has not previously been tested.

The present prospective study therefore provided, to our knowledge, the first test of the prognostic information contained in Perceived Causal Networks, examining whether such brief person‐specific representations are prospectively informative across childhood, adolescence, and early adulthood. Using both self‐ and parent‐report, we assessed PECANs at baseline and psychopathology approximately 4, 8, and 12 weeks later. We used a hierarchical approach to distinguish information contained in personalized problem composition from information contained in relational network structure. The preregistered analyses focused on characteristics of the perceived relations among problems. Exploratory post hoc analyses additionally examined problem breadth, the number of problems identified as personally relevant, as a compositional characteristic associated with subsequent psychopathology beyond baseline symptom severity. Relational characteristics were subsequently evaluated after accounting for both baseline psychopathology and problem breadth.

## METHODS

### Participants and procedure

Data were obtained from a completed longitudinal study conducted between January and August 2026 examining person‐specific representations of psychological problems in children, adolescents, and young adults. Participants were recruited through postal mailings distributed to households, a university outpatient psychotherapy clinic, schools, and social media. Depending on age, assessments were completed either by young people themselves or by a parent reporting on their child. Parent reports were obtained for children and adolescents aged 6–17 years, whereas self‐reports were obtained from adolescents and young adults aged 14–21 years. Self‐ and parent‐report data were analyzed separately because each PECAN represents the respective informant's subjective representation of the young person's problems.

At baseline (T1), participants completed a PECAN assessment and the Pediatric Symptom Checklist (PSC‐D). As in previous PECAN studies, a second PECAN assessment was administered shortly thereafter to evaluate short‐term reliability (Klintwall et al., 2023; Vogel, Fromm, et al., 2025). At this second assessment, the problems selected during the initial PECAN were retained, and participants repeated the assessment of perceived relations among these problems. Psychopathology was reassessed approximately 4, 8, and 12 weeks later (T2–T4). Reliability analyses were based on participants with sufficient repeated PECAN data. The assessment schedule is summarized in Figure 2A.

**FIGURE 2:**
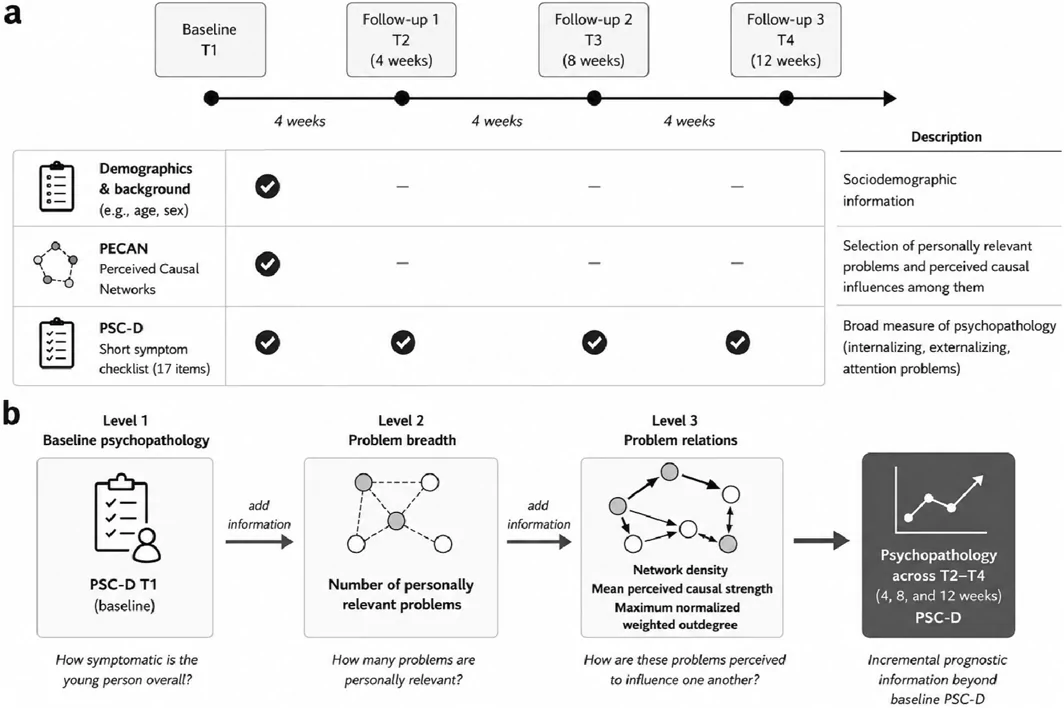
Study design and analytical approach. (A) Assessment schedule. PECAN and PSC‐D were administered at baseline (T1), with a second PECAN assessment shortly thereafter to evaluate short‐term reliability. PSC‐D psychopathology was reassessed approximately 4 weeks (T2), 8 weeks (T3), and 12 weeks (T4) later. (B) Hierarchical approach for evaluating incremental prognostic information. Baseline PSC‐D represented conventional psychopathology severity. Problem breadth, indexed by the number of individually selected problems, was first examined for prognostic information beyond baseline PSC‐D. Characteristics of problem relations—mean perceived causal strength, network density, and maximum normalized weighted outdegree—were subsequently evaluated after additional adjustment for problem breadth. Prognostic models jointly incorporated PSC‐D psychopathology across T2–T4 as the outcome. PECAN, perceived causal network; PSC‐D, pediatric symptom checklist.

The original dataset comprised 723 baseline records. Records were excluded because of duplicate participation, consent or data‐use issues, age or informant ineligibility, incomplete baseline participation, or prespecified response‐quality criteria. Detailed participant flow and reasons for exclusion are shown in Supporting Information S1: Figure S1.

The final analytic sample comprised 361 participants, including 255 self‐reports and 106 parent reports. All participants had selected at least three problems and provided sufficient baseline data to derive the relevant PECAN characteristics. The original sample size was informed by an a priori power analysis indicating that approximately 62 participants were required to detect an association of *r* = 0.35 with 80% power at *α* = 0.05; recruitment targets were increased to accommodate attrition.

The study was approved by the Ethics Committee of Johannes Gutenberg University Mainz (2025‐JGU‐psychEK‐S072). Written informed consent was obtained from legal guardians or directly from adolescents where they were considered capable of providing informed consent; assent was obtained from minors where applicable. The analyses and data‐quality procedures were preregistered on AsPredicted (No. 269,512). Reporting followed the Strengthening the Reporting of Observational Studies in Epidemiology (STROBE) guideline; the completed checklist is provided in the Appendix S1.

### Measures

#### Perceived causal networks and derived characteristics

Perceived Causal Networks are person‐specific representations of psychological problems and their perceived relations (Klintwall et al., 2023; Vogel, Blanken, et al., 2025). Participants selected 3–7 personally relevant problems from a 24‐item transdiagnostic problem pool. The pool was developed as a clinically and developmentally informed set of candidate problems intended to provide broad coverage of psychological difficulties relevant to children and adolescents while remaining suitable for use in a community‐based sample. HiTOP served as a conceptual framework for broad transdiagnostic coverage rather than as a formal one‐to‐one mapping of individual problems onto HiTOP spectra. The pool additionally included developmentally relevant interpersonal, school‐related, behavioral, and health‐related difficulties that may be salient to young people and parents but are not fully represented by conventional symptom categories. Short problem labels were accompanied by informant‐adapted explanatory descriptions intended to facilitate comprehension. The range of 3–7 problems was chosen to provide a minimally informative relational representation while maintaining feasibility and reliability, consistent with previous PECAN‐CA findings (Vogel, Fromm, et al., 2025). The problem list covered internalizing, externalizing, attentional, behavioral, cognitive, interpersonal, somatic, and health‐related difficulties and is provided in Supporting Information S1: Table S1.

For each selected problem, participants rated current severity from 0 (*no distress*) to 10 (*extreme distress*). Perceived relations were subsequently elicited for each selected problem. Participants were instructed to consider: “If this difficulty gets worse, which other difficulties also get worse?” and “What else can this difficulty lead to for you?” They then indicated which of their other selected problems were influenced by the focal problem. For each endorsed relation, participants rated its strength from 1 to 10, with the endpoints labeled “very low influence” and “very strong influence.” The resulting PECAN is therefore a directed, weighted representation of participants' subjective understanding of how their problems influence one another rather than evidence of objective causal effects.

The core PECAN network‐construction component—selection of personally relevant problems, severity ratings, and ratings of perceived relations—was timed electronically. Completion time was calculated by summing the corresponding page‐level completion times; values exceeding 30 min were excluded from timing descriptives as likely interruptions.

We derived characteristics corresponding to three complementary aspects of the individualized problem representation: ***Problem breadth*** was the number of problems selected as personally relevant (range 3–7). All selected problems contributed to this count irrespective of their subsequent severity rating, because problem selection and current severity were treated as distinct components of the personalized representation. ***Problem intensity*** was indexed by mean severity across selected problems. Summed PECAN severity, used in preregistered concurrent analyses, combined breadth and intensity and was therefore not interpreted as a breadth‐independent intensity measure. Three characteristics summarized ***problem relations***. Mean perceived causal strength was the average strength of endorsed relations. Network density was the proportion of all possible directed relations that were endorsed. Weighted outdegree was the summed strength of outgoing relations for each problem, normalized by its maximum possible value, 10 (*k* − 1); the maximum normalized weighted outdegree across problems was retained.

#### Pediatric Symptom Checklist

General psychopathology was assessed using the 17‐item German Pediatric Symptom Checklist (PSC‐D; Dippel et al., 2026) in corresponding self‐ and parent‐report versions. Items are rated from 0 (*never*) to two (*often*), yielding total scores from 0 to 34. The total score was used at baseline and as the primary follow‐up outcome; internalizing and externalizing subscales were examined exploratorily. Self‐report PECAN characteristics were related to self‐reported PSC‐D scores and parent‐report characteristics to parent‐reported scores.

The German PSC‐D has been validated in children and adolescents up to age 17 (Dippel et al., 2026). Because the self‐report sample also included young adults aged 18–21 years, central self‐report analyses were repeated after restricting the sample to the age range covered by the German validation. The PSC‐17 has previously been used in samples including participants up to age 21 (Doupnik et al., 2017).

Internal consistency of the PSC‐D total score was evaluated separately by informant and measurement occasion.

### Statistical analysis

Self‐ and parent‐report data were analyzed separately throughout. Unless otherwise stated, continuous PECAN predictors were standardized within informant group, effect estimates are reported with 95% confidence intervals, and two‐sided statistical tests were evaluated at *α* = 0.05.

#### Reliability and concurrent associations

Following previous PECAN studies (Klintwall et al., 2023; Vogel, Fromm, et al., 2025), short‐term reliability was assessed using within‐person Spearman correlations for weighted directed relations and absolute‐agreement intraclass correlation coefficients for weighted outdegree. For relation‐level reliability, participant‐specific Spearman correlations were calculated between the complete weighted relation vectors of the two PECAN assessments, with absent relations represented by zero; the median correlation and a bootstrap 95% confidence interval were used as summary measures. Outdegree reliability was quantified using a two‐way absolute‐agreement, single‐measure ICC [ICC(A, 1)].

Concurrent associations between PECAN characteristics and baseline PSC‐D psychopathology were examined separately by informant. Pearson correlations were used as primary estimates, with Spearman correlations examined as rank‐based sensitivity analyses. Mean severity across selected problems was examined as the breadth‐independent indicator of problem intensity. The preregistered analysis using summed PECAN severity was additionally retained; because this score combines the number and severity of selected problems, it was interpreted as an indicator of combined problem breadth and intensity rather than problem intensity alone. Mean perceived causal strength and network density were examined as characteristics of problem relations. Their joint associations of causal strength and density with baseline psychopathology were additionally examined using standardized linear regression models.

#### Incremental prognostic analyses

The central analyses examined whether baseline person‐specific characteristics provided prognostic information beyond baseline psychopathology. All available PSC‐D observations at T2–T4 were analyzed jointly using linear mixed‐effects models with participant‐specific random intercepts. Models included baseline PSC‐D total score and follow‐up time, coded 0, 1, and 2 for T2, T3, and T4, respectively. Continuous PECAN predictors were standardized within informant group.

Analyses followed a hierarchical approach (Figure 2B). Problem breadth, indexed by the number of selected problems, was first examined after adjustment for baseline PSC‐D and follow‐up time. Problem breadth had not been specified as the central prognostic construct in the preregistration and was therefore treated as an exploratory post hoc characteristic of the personalized problem representation. Characteristics of problem relations, mean perceived causal strength, network density, and maximum normalized weighted outdegree, were subsequently examined in separate models additionally adjusted for problem breadth. Maximum normalized weighted outdegree was likewise added post hoc as an exploratory relational characteristic.

Incremental model fit associated with problem breadth was evaluated using likelihood‐ratio tests and changes in Akaike and Bayesian information criteria. Benjamini–Hochberg false‐discovery‐rate correction was applied across the three relational characteristics separately within each informant group. Problem breadth was treated as a distinct conceptual test and was therefore not included in this correction.

The preregistered prospective analysis plan included longitudinal trajectory models and baseline‐adjusted prospective analyses, with the latter originally framed as sensitivity analyses. We emphasize the baseline‐adjusted models in the present manuscript because they most directly address whether PECAN characteristics provide prognostic information beyond current psychopathology. The preregistered trajectory analyses are reported in the Supporting Information S1: Table S5. All analytic refinements relative to the preregistration—including the post hoc examination of problem breadth and maximum normalized weighted outdegree—are summarized in Supporting Information S1: Table S2. Detailed supporting analyses are reported in Supporting Information S1: Table S3 (retest reliability), Table S4a,b (concurrent associations), Table S6a–c (sensitivity analyses), Supporting Information S1: Table S7 (attrition analyses), and Supporting Information S1: Table S8 (exploratory subscale analyses).

#### Sensitivity and exploratory analyses

Sensitivity analyses examined robustness to source subsample, follow‐up availability, conventional PSC‐D item breadth, response quality, and restriction of self‐report analyses to ages 14–17 years. Pediatric Symptom Checklist item breadth was defined as the number of baseline items endorsed at least *sometimes* (>0). Internalizing and externalizing PSC‐D scores were examined as exploratory secondary outcomes. Further details are provided in Supporting Information S1: Table S8.

A post hoc sensitivity analysis examined whether the prognostic association of problem breadth depended on including selected problems with very low current severity. The central models were therefore repeated using a severity‐qualified breadth score counting only selected problems rated ≥2 on the 0–10 severity scale. This threshold was used as a reviewer‐motivated robustness check rather than as a validated clinical cutoff.

## RESULTS

### Sample characteristics and follow‐up

The final analytic sample comprised 361 participants, including 255 self‐report and 106 parent‐report participants. Self‐report participants were 14–21 years old (*M* = 18.14, SD = 1.69), whereas young people represented in the parent‐report sample were 6–17 years old (*M* = 10.14, SD = 3.07). Baseline PSC‐D scores averaged 9.49 (SD = 5.71) in self‐report and 11.67 (SD = 5.55) in parent‐report. For descriptive context, both mean scores were below the screening threshold of 15 established for the original PSC‐17 (Gardner et al., 1999). This threshold indicates a positive psychosocial screen rather than a diagnostic or clinical severity classification. German norms distinguishing normal, subclinical, and clinical ranges have not yet been established for the PSC‐D and are currently under development (Dippel et al., 2026). Participants selected on average 4.42 (SD = 1.68) and 4.30 (SD = 1.54) personally relevant problems, respectively. Mean problem intensity was 7.40 (SD = 1.86) in self‐report and 6.67 (SD = 1.86) in parent‐report. Further baseline characteristics are presented in Table 1. Internal consistency of the PSC‐D total score was acceptable to good across measurement occasions in both self‐report (Cronbach's *α* = 0.729–0.835) and parent‐report (*α* = 0.756–0.783). The core PECAN network construction took on average 3.96 min (SD = 3.08; median = 3.26; IQR = 1.78–5.20) among 332 participants with usable timing data.

**TABLE 1:** Baseline sample and PECAN characteristics.

Characteristic	Self‐report (*n* = 255)	Parent‐report (*n* = 106)
Age, years, *M* (SD), range	18.14 (1.69), 14–21	10.14 (3.07), 6–17
Female, *n* (%)	166 (65.1)	52 (49.1)
Male, *n* (%)	86 (33.7)	53 (50.0)
Diverse, *n* (%)	3 (1.2)	1 (0.9)
PSC‐D total at baseline, *M* (SD)	9.49 (5.71)	11.67 (5.55)
Problem breadth: Number of selected problems, *M* (SD), range	4.42 (1.68), 3–7	4.30 (1.54), 3–7
Problem intensity: Mean severity, *M* (SD)	7.40 (1.86)	6.67 (1.86)
Mean perceived causal strength, *M* (SD)	7.44 (1.77)	6.80 (1.90)
Network density, *M* (SD)	0.48 (0.12)	0.49 (0.16)
Maximum normalized weighted outdegree, *M* (SD)	0.44 (0.13)	0.44 (0.15)

Abbreviation: PECAN, perceived causal network.

Follow‐up PSC‐D data were available for 231 participants at T2, 179 at T3, and 172 at T4. Overall, 235 participants (65.1%) contributed at least one follow‐up assessment, including 161 self‐report participants (63.1%) and 74 parent‐report participants (69.8%); 163 participants completed all four assessments.

Follow‐up availability was associated with several baseline characteristics, including problem breadth in the parent‐report sample and, for T4 availability, in the self‐report sample. Additional associations were observed for baseline psychopathology, age, characteristics of problem relations, and source subsample. Primary mixed‐effects models incorporated all available follow‐up observations rather than requiring complete cases.

### Reliability and concurrent associations

Short‐term reliability of weighted problem relations differed between informants. Median within‐person Spearman correlations between the two PECAN assessments were high in self‐report (median *ρ* = 0.984, 95% CI [0.763, 1.00]) and moderate in parent‐report (median *ρ* = 0.518, 95% CI [0.402, 0.645]). Weighted outdegree showed ICCs of 0.785 (95% CI [0.718, 0.837]) in self‐report and 0.732 (95% CI [0.684, 0.773]) in parent‐report; thus, the preregistered ICC threshold of 0.80 was not reached in either informant group. Detailed reliability analyses are reported in Supporting Information S1: Figure S2 and Table S3.

Concurrent associations with baseline psychopathology differed across characteristics of the person‐specific representation. Summed PECAN severity, which combines problem breadth and intensity, was positively associated with baseline PSC‐D scores in both self‐report (*r* = 0.589, *p* < 0.001) and parent‐report (*r* = 0.262, *p* = 0.007). In contrast, mean severity across selected problems showed no positive association with baseline PSC‐D scores in self‐report (*r* = −0.121, *p* = 0.054) or parent‐report (*r* = −0.167, *p* = 0.088).

Characteristics of perceived problem relations showed small inverse concurrent associations with psychopathology. Mean perceived causal strength was negatively associated with baseline PSC‐D scores in both self‐report (*r* = −0.186, *p* = 0.003) and parent‐report (*r* = −0.257, *p* = 0.008). Network density was negatively associated with PSC‐D scores in self‐report (*r* = −0.211, *p* < 0.001), but not in parent‐report (*r* = −0.088, *p* = 0.370). In models including both relational characteristics, causal strength and density retained independent associations in self‐report (*β* = −0.144, *p* = 0.023; *β* = −0.176, *p* = 0.005, respectively), whereas only causal strength remained associated in parent‐report (*β* = −0.249, *p* = 0.011; density: *β* = −0.050, *p* = 0.602). Full concurrent analyses are provided in Supporting Information S1: Table S4a,b.

### Incremental prognostic information of problem breadth

Problem breadth provided prognostic information beyond baseline psychopathology in both informant groups. As expected, baseline PSC‐D strongly predicted subsequent psychopathology (self‐report: *b* = 0.61, 95% CI [0.52, 0.70], *p* < 0.001; parent‐report: *b* = 0.60, 95% CI [0.49, 0.71], *p* < 0.001). After adjustment for baseline PSC‐D and follow‐up time, greater problem breadth was associated with higher PSC‐D scores across T2–T4 in self‐report (*b* = 1.23, 95% CI [0.67, 1.79], *p* < 0.001) and parent‐report (*b* = 1.48, 95% CI [0.78, 2.17], *p* < 0.001).

Thus, individuals who identified a broader range of problems as personally relevant showed higher subsequent psychopathology even when they had comparable levels of psychopathology at baseline. A 1‐*SD* increase in problem breadth corresponded to approximately 1.68 additional selected problems in self‐report and 1.54 in parent‐report. Expressed per additional selected problem, this corresponds to approximately 0.73 higher PSC‐D points in self‐report and 0.96 higher PSC‐D points in parent‐report.

Adding problem breadth improved model fit beyond models containing baseline PSC‐D and follow‐up time alone in both self‐report (ΔAIC = −15.50; ΔBIC = −11.51; likelihood‐ratio *p* < 0.001) and parent‐report (ΔAIC = −13.32; ΔBIC = −10.11; likelihood‐ratio *p* < 0.001).

### Problem relations beyond problem breadth

In contrast to problem breadth, characteristics of perceived problem relations provided little consistent additional prognostic information. After adjustment for baseline PSC‐D, follow‐up time, and problem breadth, mean perceived causal strength was not associated with subsequent psychopathology in either self‐report (*b* = 0.39, 95% CI [−0.03, 0.82], *p* = 0.071) or parent‐report (*b* = −0.39, 95% CI [−1.09, 0.31], *p* = 0.275). Maximum normalized weighted outdegree was likewise not associated with subsequent PSC‐D scores (self‐report: *b* = −0.26, 95% CI [−0.68, 0.15], *p* = 0.216; parent‐report: *b* = −0.38, 95% CI [−1.15, 0.40], *p* = 0.338).

Network density showed an inverse association with subsequent psychopathology in self‐report (*b* = −0.59, 95% CI [−1.07, −0.11], *p* = 0.017), but not in parent‐report (*b* = −0.38, 95% CI [−1.13, 0.38], *p* = 0.328). The self‐report density association was at the Benjamini–Hochberg false‐discovery‐rate threshold across the three relational characteristics (*q* ≈ 0.050) and was therefore interpreted cautiously. Overall, relational characteristics did not show a consistent pattern of incremental prognostic associations across informants. The network‐density associations are illustrated in Figure 3.

**FIGURE 3:**
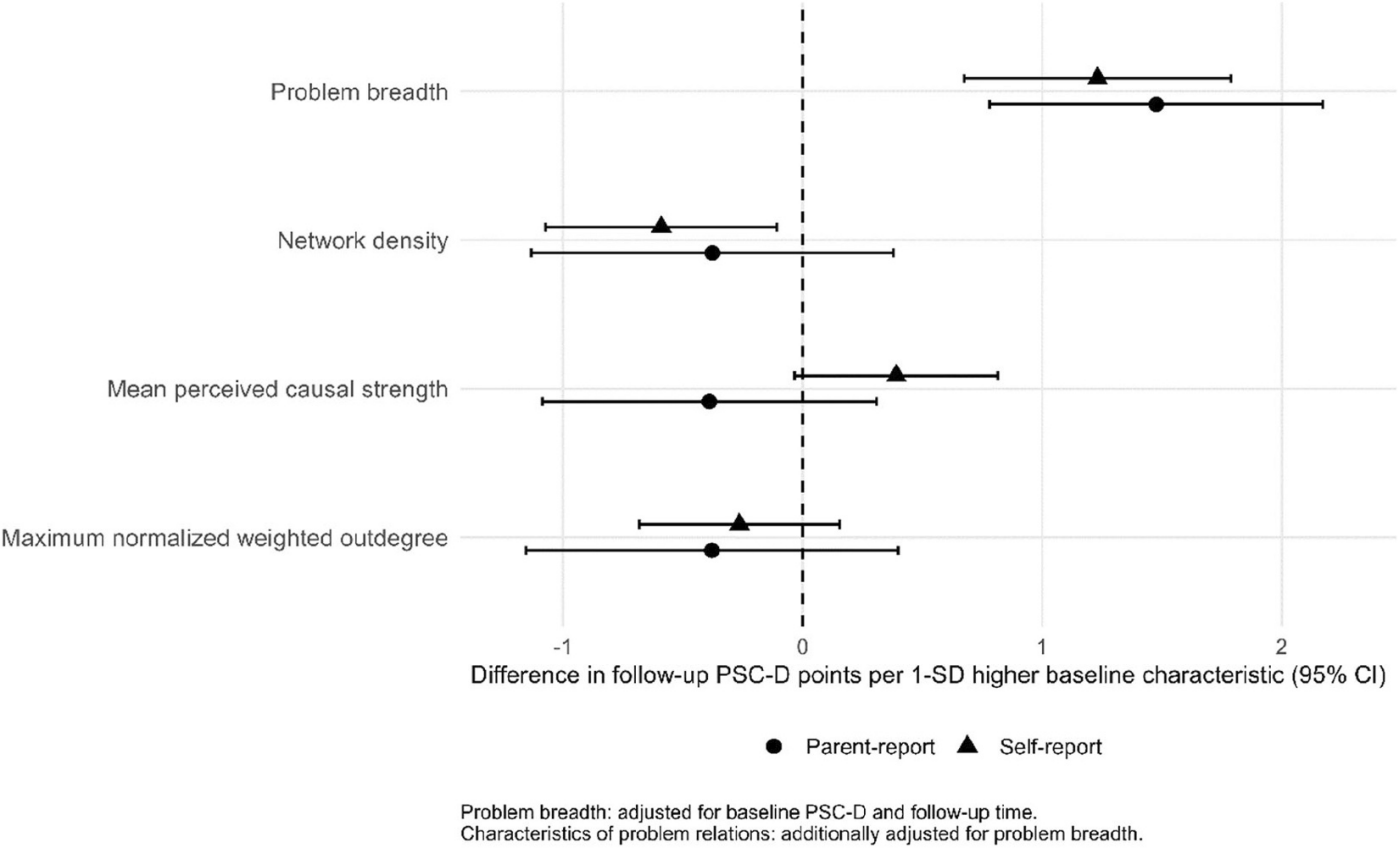
Incremental prognostic associations of baseline person‐specific problem characteristics with subsequent psychopathology. Points represent regression coefficients from linear mixed‐effects models for PSC‐D scores across T2–T4 per 1‐SD higher baseline characteristic; lines indicate 95% confidence intervals. Problem breadth was adjusted for baseline PSC‐D and follow‐up time. Characteristics of problem relations—network density, mean perceived causal strength, and maximum normalized weighted outdegree—were additionally adjusted for problem breadth. Triangles represent self‐report and circles parent‐report estimates. The dashed vertical line indicates no association. PSC‐D, pediatric symptom checklist.

### Sensitivity and preregistered longitudinal analyses

The association between problem breadth and subsequent psychopathology was robust across sensitivity analyses. Estimates were essentially unchanged after additional adjustment for the number of baseline PSC‐D items endorsed at least *sometimes* (self‐report: *b* = 1.22, 95% CI [0.66, 1.77], *p* < 0.001; parent‐report: *b* = 1.53, 95% CI [0.83, 2.22], *p* < 0.001). Perceived Causal Network problem breadth was moderately associated with PSC‐D item breadth in self‐report (*r* = 0.61) and more weakly in parent‐report (*r* = 0.39), indicating overlap but not equivalence between personalized problem breadth and breadth of symptom endorsement on the conventional measure.

The problem‐breadth association was also unchanged when only selected problems with severity ratings ≥2 were counted (self‐report: *b* = 1.27, 95% CI [0.72, 1.83], *p* < 0.001; parent‐report: *b* = 1.48, 95% CI [0.78, 2.17], *p* < 0.001). Only 18 of 1584 selected problems (1.1%) had severity ratings below this threshold.

Adjustment for source subsample likewise produced little change in the problem‐breadth estimates (self‐report: *b* = 1.25, 95% CI [0.66, 1.85], *p* < 0.001; parent‐report: *b* = 1.47, 95% CI [0.77, 2.18], *p* < 0.001), with similar results when source‐by‐time interactions were included. Excluding participants with a Response Speed Index >2 likewise produced little change in the central findings.

Restricting self‐report analyses to participants aged 14–17 years left 22 participants with data available across all measurement occasions (T1–T4). Despite the small sample, problem breadth remained positively associated with subsequent psychopathology (*b* = 1.51, *p* = 0.045). In contrast, the inverse density association observed in the full self‐report sample was not reproduced in this age‐restricted analysis (*b* = 0.34, *p* = 0.529), further indicating limited robustness of the relational characteristics.

The preregistered trajectory analyses similarly provided little evidence that baseline characteristics of problem relations were consistently associated with differential change in psychopathology over time. An inverse time‐by‐density association emerged in self‐report but was not robust across sensitivity analyses or reproduced in parent‐report. Preregistered trajectory analyses are reported in Supporting Information S1: Table S5; sensitivity analyses, including response‐speed and age‐restricted analyses, are reported in Table S6a–c; attrition analyses are reported in Supporting Information S1: Table S7; and exploratory subscale analyses are reported in Supporting Information S1: Table S8.

## DISCUSSION

The present study addressed a foundational question for the use of person‐specific networks in personalized assessment and psychotherapy: which information contained in an individual's network is prospectively informative beyond conventional symptom severity? To our knowledge, this is the first prospective evaluation of Perceived Causal Networks. The central finding was that the relational component of PECANs showed little consistent incremental prognostic information. Mean perceived causal strength and maximum normalized weighted outdegree were not associated with subsequent psychopathology, and the inverse association observed for network density in self‐report was neither reproduced in parent‐report nor robust across sensitivity analyses. Thus, the aspects of PECANs most directly reflecting their network structure did not consistently predict subsequent psychopathology beyond baseline severity and problem breadth. In contrast, exploratory post hoc analyses indicated that problem breadth—the number of problems considered personally relevant—was prospectively informative in both self‐ and parent‐report. This pattern suggests that the most consistent prognostic information in the present study resided in the composition of the personalized problem representation rather than in its perceived relational structure.

This distinction reflects two conceptually different components of PECANs: individuals first define which problems constitute their personalized representation and subsequently specify relations among them (Vogel, Blanken, et al., 2025). The present findings suggest that, within this brief single‐assessment format, these components do not contribute equally to prospective information. The distinction between compositional and relational information is also important when considering the validity of PECANs. Respondents may be able to reliably identify and prioritize the problems they experience while having more limited insight into how these problems influence one another (Vogel, Blanken, et al., 2025). Accordingly, reproducibility of perceived causal relations should not be interpreted as evidence that these relations correspond to causal processes operating over time. Although the endpoints of the causal‐strength scale were verbally labeled, ratings were not anchored to concrete frequencies, situations, or external reference points. Individual differences in scale use may therefore have contributed to between‐person differences in relational characteristics based on edge strength. Future work should examine alternative causal phrasings and more concrete or comparative anchors, compare subjective relations with EMA‐derived temporal relations, and test whether clinician‐assisted elicitation, integration of empirical prior knowledge, or combination of information across informants improves the validity of relational judgments (Scholten et al., 2025; Vogel, Blanken, et al., 2025). The marked difference in relation‐level reliability between self‐ and parent‐report should also be interpreted cautiously because informant perspective was confounded with age: self‐reports were obtained from adolescents and young adults, whereas parent reports predominantly represented younger children and adolescents.

Several non‐mutually exclusive explanations may account for the association with problem breadth. First, breadth may capture a compositional aspect of psychopathology not fully represented by aggregate severity. Identical sum scores can correspond to markedly different symptom profiles (Fried & Nesse, 2015), and a broader personally relevant problem repertoire may reflect a more multifaceted or less circumscribed presentation. This interpretation is consistent with longitudinal findings linking higher symptom counts and difficulties spanning multiple domains to poorer subsequent outcomes (Vander Stoep et al., 2002; Verhulst & van der Ende, 1993). The present study extends this work by showing that personalized problem breadth remained prospectively informative even when baseline psychopathology was held constant.

Importantly, the breadth association also remained essentially unchanged after additional adjustment for the number of PSC‐D items endorsed at baseline. Perceived Causal Network problem breadth was therefore related to, but not interchangeable with, conventional symptom breadth. One possible explanation is that personalized problem selection captures difficulties that fall partly outside the content of a brief fixed‐item questionnaire. Perceived Causal Network allowed participants to select from a broad transdiagnostic range of problems informed by HiTOP (Kotov et al., 2021), whereas the PSC‐D comprises 17 fixed items (Dippel et al., 2026). At the same time, the present findings do not establish that personalized selection is necessary to recover this information. A more comprehensive fixed transdiagnostic assessment might capture similar breadth. Accordingly, the present breadth finding should not be interpreted as evidence that personalization itself accounts for the incremental prognostic information. The effect may partly reflect the broader or different content sampled by the PECAN problem pool relative to the PSC‐D. Direct comparisons will therefore be important for determining whether the observed signal reflects personalization itself, broader content coverage, or both.

Problem breadth may additionally reflect how individuals perceive and prioritize their difficulties. PECANs do not simply record endorsement of predefined symptoms; respondents identify which problems they consider personally relevant (Vogel, Blanken, et al., 2025). Breadth may therefore capture the perceived salience or scope of an individual's difficulties before any relational network is constructed. Because PECAN characteristics and PSC‐D outcomes were obtained from the same informant, shared reporting tendencies cannot be excluded. Informant agreement in young people varies with factors such as problem salience and observability (Karver, 2006). Nevertheless, the replication of the breadth association across self‐ and parent‐report is noteworthy given the different perspectives from which young people's difficulties were represented.

The comparatively limited prognostic information contained in relational characteristics is equally relevant for evaluating person‐specific networks. Previous studies in adults have suggested that statistically estimated person‐specific symptom relations can contribute to prediction of psychotherapy outcome and later symptom course (Cusack et al., 2024; Schumacher et al., 2024, 2026). These findings differ from the present pattern, but the approaches capture fundamentally different forms of person‐specific information. Statistically estimated longitudinal networks derive relations from repeated within‐person observations, whereas PECAN relations represent respondents' subjective perceptions of how their problems influence one another within a single assessment (Klintwall et al., 2023; Vogel, Blanken, et al., 2025). The present results should therefore not be interpreted as evidence against relational approaches more generally.

Moreover, our analyses summarized relational organization using global characteristics such as mean perceived causal strength, density, and maximum outdegree. Global summary characteristics may not fully capture the aspects of relational organization most relevant to the network hypothesis (Bringmann, 2021; Cusack et al., 2025). Relevant information may instead reside in particular relations, local structures, or the identity of specific highly connected problems. Idiographic network characteristics can vary substantially depending on symptom selection and observation period (Cusack et al., 2025), and a relation that is clinically meaningful for one individual need not translate into a prognostically informative global network statistic across individuals. Future studies should therefore compare alternative representations of relational structure rather than interpreting the present null findings as a definitive test of the prognostic relevance of network organization. The inverse density association observed in self‐report was not reproduced in parent‐report or the age‐restricted sensitivity analysis and should therefore be interpreted cautiously. Overall, the present results indicate limited incremental prognostic value of the global relational characteristics examined here rather than an absence of potentially meaningful relational information within PECANs.

These findings are relevant to personalized assessment and psychotherapy in young people. Personalized interventions show a small advantage over standardized approaches in adolescents, although implementation varies considerably (Li et al., 2024). Person‐specific networks represent one possible route to such personalization and are increasingly proposed for case conceptualization and treatment targeting (Bringmann, 2021; Burger et al., 2022; Vogel, Blanken, et al., 2025; von Klipstein et al., 2020; Wright & Woods, 2020). The present study addresses one prerequisite for these applications—prospective validity—and suggests that simple problem breadth may carry more consistent information than global relational characteristics. Direct comparisons with previous PECAN studies are difficult because assessment formats and timing definitions differed. The original online PECAN required a median of 22.7 minutes including immediate retest items, whereas the interviewer‐guided PECAN‐CA procedure required approximately 29 minutes on average including 10 minutes of psychoeducation (Klintwall et al., 2023; Vogel, Fromm, et al., 2025). The present estimate of 3.26 minutes refers only to the core network‐construction component and to relatively small networks constrained to 3–7 selected problems. The resulting networks were therefore deliberately brief representations rather than exhaustive mappings of participants' difficulties. Accordingly, the present completion time should not be interpreted as a direct reduction relative to earlier PECAN estimates. Nevertheless, such brief completion raises the question of how much reflection participants devoted to perceived causal relations. The substantial short‐term consistency of relational ratings, particularly in self‐report, argues against an explanation based solely on careless responding, but does not establish the validity of these judgments.

Several limitations should be considered. First, problem breadth was intentionally constrained to between three and seven selected problems as part of the PECAN procedure. The present measure therefore captures variation in the breadth of an individual's prioritized problem representation rather than an exhaustive count of all psychological difficulties. Although problem labels were accompanied by informant‐adapted explanatory descriptions, comprehension of the individual problem terms was not formally assessed in the present study. This is relevant because previous PECAN‐CA findings suggest that understanding of symptom and network concepts may affect the reliability and feasibility of the resulting representations (Vogel, Fromm, et al., 2025). Second, PECAN characteristics and PSC‐D outcomes were assessed from the same informant, leaving open the possibility of shared perception or reporting effects. Replication using cross‐informant, clinician‐rated, or functional outcomes would provide stronger evidence of external validity. Third, follow‐up participation declined across the 12‐week study period and was associated with some baseline characteristics. Although the mixed‐effects models incorporated all available follow‐up observations and the central breadth association was robust across multiple sensitivity analyses, bias due to informative missingness cannot be excluded. Fourth, the follow‐up period was relatively short, and the findings therefore concern short‐term course rather than longer‐term developmental or treatment outcomes.

Finally, the present study examined incremental prognostic associations rather than developing and externally validating an individual‐level prediction model. The findings demonstrate that problem breadth contains information about subsequent psychopathology beyond baseline severity, but they do not establish predictive accuracy, decision utility, or benefit from network‐informed treatment selection for individual patients. Likewise, the limited findings for relational characteristics cannot exclude prognostic or therapeutic value contained in specific relations, local network structures, or alternative representations of relational information. Independent replication in larger longitudinal and clinical samples, longer follow‐up periods, and ultimately prospective evaluations of network‐informed clinical decisions will be required to establish which components of person‐specific networks are most useful for personalized psychotherapy.

## CONCLUSION

Across childhood, adolescence, and early adulthood, a brief person‐specific assessment contained prospective information beyond conventional baseline psychopathology in both self‐ and parent‐report. Importantly, this information was not distributed uniformly across components of the personalized network. Exploratory analyses indicated that the most consistent signal was contained in problem breadth: young people whose representations encompassed a broader range of personally relevant difficulties showed higher subsequent psychopathology despite comparable baseline symptom severity. In contrast, global characteristics of perceived relational structure provided little consistent additional information beyond problem breadth. These findings provide a first prospective evaluation of PECANs and underscore the importance of evaluating compositional and relational components of person‐specific models separately. Confirmatory replication is needed before either component is used to inform personalized clinical decisions.

## AUTHOR CONTRIBUTIONS

**Felix Vogel**: Conceptualization; investigation; writing—original draft; writing—review and editing; visualization; validation; methodology; formal analysis; project administration; data curation; supervision; resources. **Stefanie Jungmann**: Resources; writing—review and editing; supervision.

## CONFLICT OF INTEREST STATEMENT

The authors declare no conflict of interest.

## ETHICAL CONSIDERATIONS

The study was approved by the Ethics Committee of Johannes Gutenberg University Mainz on December 19, 2025 (reference no. 2025‐JGU‐psychEK‐S072). Written informed consent was obtained from legal guardians or directly from adolescents where they were considered capable of providing informed consent; assent was obtained from minors where applicable.

## Supporting information

Supporting Information S1

## Data Availability

The individual‐level data generated and analyzed in this study are not publicly available due to ethical and data‐protection restrictions. Additional information regarding the data and analyses is available on request from the corresponding author and subject to applicable ethical and legal requirements. Analysis code is available at: https://osf.io/7ekjd/overview.
