# Supplementary material for: Beyond symptom severity: Prognostic information from subjective person‐specific problem networks in young people
Source: JCPP Adv. 2026 Nov 11:e70189. Online ahead of print. doi: 10.1002/jcv2.70189 (PMC13752686; doi:10.1002/jcv2.70189)
Supplement: Supplementary file 1 [file JCV2-9999-e70189-s001.docx]

**Beyond Symptom Severity: Prognostic Information From Subjective Person-Specific Problem Networks in Young People**

**Supporting Information**

**Figure S1. Participant flow.** Flow from 723 raw baseline records to the final analytic sample of 361 participants (255 self-reports and 106 parent reports). Records were sequentially screened for duplicate participation, consent and permission for data use, age and informant eligibility, completion of the baseline assessment, and the prespecified completion-time criterion. Pediatric Symptom Checklist (PSC) follow-up data were available for 231 participants at T2 (approximately 4 weeks), 179 at T3 (approximately 8 weeks), and 172 at T4 (approximately 12 weeks); 235 participants provided at least one post-baseline PSC assessment.

**Figure S2.** **Short-term PECAN retest reliability.** Short-term reliability of weighted directed relations was summarized using the median within-person Spearman correlation between the two PECAN assessments, with 95% bootstrap confidence intervals. Reliability of weighted outdegree was assessed using a two-way absolute-agreement, single-measure intraclass correlation coefficient [ICC(A,1)]. Triangles represent self-report and circles parent-report estimates. Dashed vertical lines indicate the preregistered thresholds of ρ = .50 for weighted relation correspondence and ICC = .80 for outdegree reliability.

**Table S1. Transdiagnostic PECAN problem list.**

| **No.** | **English conceptual label** | **German short label** |
| --- | --- | --- |
| 1 | Worry | Sorgen |
| 2 | Rumination | Grübeln |
| 3 | Negative thoughts | Negative Gedanken |
| 4 | Difficulties managing emotions | Schwierigkeiten im Umgang mit Gefühlen |
| 5 | Anxiety and insecurity | Ängstlichkeit und Unsicherheit |
| 6 | Sad or depressed mood | Traurige oder gedrückte Stimmung |
| 7 | Irritability and inner tension | Reizbarkeit und innere Anspannung |
| 8 | Low self-confidence | Geringes Selbstvertrauen |
| 9 | Self-harm | Selbstverletzung |
| 10 | Problems with peers | Probleme mit Gleichaltrigen |
| 11 | Social withdrawal | Sozialer Rückzug |
| 12 | Aggressive behavior | Aggressives Verhalten |
| 13 | Problems with rules / rule-breaking | Probleme mit Regeln |
| 14 | Marked restlessness or urge to move | Starke Unruhe oder Bewegungsdrang |
| 15 | Acting without thinking / impulsive behavior | Unüberlegtes Handeln |
| 16 | Concentration difficulties | Konzentrationsprobleme |
| 17 | Physical complaints | Körperliche Beschwerden |
| 18 | Sleep problems | Schlafprobleme |
| 19 | Obsessive thoughts or compulsive behaviors | Zwanghafte Gedanken oder Handlungen |
| 20 | Avoiding school or training | Schule oder Ausbildung meiden |
| 21 | Eating problems | Probleme mit dem Essen |
| 22 | Problematic media use | Problematischer Medienkonsum |
| 23 | Procrastination | Aufschieben von Aufgaben |
| 24 | Low physical activity | Wenig körperliche Bewegung |

**Note.** The full study materials contained informant-adapted explanatory wording for each problem; the table reproduces the short problem labels used to define the item pool.

**Table S2. Preregistration, analytic implementation, and transparent refinements.**

| **Element** | **Preregistered / planned element** | **Final implementation and rationale** |
| --- | --- | --- |
| Weighted relation retest | Within-person Spearman correspondence of weighted directed relations | Conducted as preregistered; median participant-level ρ used as summary. |
| Outdegree retest | Absolute-agreement ICC for weighted outdegree | Implemented as two-way absolute-agreement, single-measure ICC(A,1), matching the intended reliability question. |
| Number of selected problems | Preregistered minimum of 3, maximum of 7 | The PECAN interface allowed 3–7 problems. |
| Reliability as data-quality criterion | Retest reliability considered as a possible quality indicator | No participant was excluded based on low retest reliability because reliability was itself an outcome of RQ1. |
| Fast completion | Exclude implausibly fast completion | Implemented as TIME_SUM < one third of the source-specific median after requiring FINISHED = 1. |
| Attention check | Planned data-quality exclusion | Applied as preregistered based on a single item in the questionnaire. |
| PECAN problem severity | Summed severity | Summed severity retained for the preregistered concurrent analysis; mean severity added as a breadth-independent measure of problem intensity. |
| Mean causal strength | Average causal-strength parameter | Operationalized as the mean strength among endorsed relations, separating relation magnitude from density; an all-edge mean was treated only as an operationalization sensitivity. |
| Prospective trajectory analysis | T1–T4 time × PECAN characteristic | Conducted and fully retained as the preregistered trajectory analysis. |
| Baseline-adjusted prognostic model | Baseline-adjusted analyses anticipated as sensitivity | Promoted to the central manuscript analysis because the T2–T4 baseline-adjusted model directly addresses incremental prognostic information beyond current psychopathology. |
| Problem breadth | Not specified as the central prognostic construct | Added post hoc/exploratorily as a theoretically interpretable compositional characteristic: number of personally relevant problems. |
| Maximum normalized weighted outdegree | Not specified as prognostic predictor | Added post hoc/exploratorily to examine dominant outgoing influence while normalizing for network size. |
| Source adjustment | Not part of primary model | Added as a sensitivity analysis because source datasets differed in composition and follow-up availability. |
| PSC-D subscales | Secondary analyses | Internalizing and externalizing scores analyzed as exploratory secondary outcomes; PSC-D total remained the central outcome. |
| PSC-D item breadth | Not preregistered | Single targeted post-hoc robustness analysis added after coauthor review. It tests whether PECAN problem breadth remains informative beyond a count of endorsed items on the fixed 17-item PSC-D. No alternative endorsement thresholds were tested. |

**Note.** The PSC-D item-breadth sensitivity was performed after the original analysis freeze as a targeted robustness analysis motivated by coauthor review and is explicitly labeled post hoc.

**Table S3. Short-term PECAN retest reliability.**

| **Informant** | **Reliability analysis** | **Estimate** | **95% CI** | **Preregistered threshold** | **Interpretation** |
| --- | --- | --- | --- | --- | --- |
| Self-report | Weighted directed relations: primary complete-vector analysis | .984 | [.763, 1.000] | >.50 | Threshold met; 74.4% of estimable individual ρ values > .50 |
| Parent-report | Weighted directed relations: primary complete-vector analysis | .518 | [.402, .645] | >.50 | Threshold met; 52.0% of estimable individual ρ values > .50 |
| Self-report | Binary relation-presence sensitivity | .882 | [.707, 1.000] | >.50 | Exploratory sensitivity; 69.5% of estimable individual ρ values > .50 |
| Parent-report | Binary relation-presence sensitivity | .481 | [.375, .707] | >.50 | Exploratory sensitivity; median below threshold |
| Self-report | Pairwise-available weighted-relation sensitivity | .783 | [.700, .949] | >.50 | Exploratory sensitivity; 69.5% of estimable individual ρ values > .50 |
| Parent-report | Pairwise-available weighted-relation sensitivity | .518 | [.402, .645] | >.50 | Same estimate as primary because A2 weights were complete |
| Self-report | Weighted outdegree: ICC(A,1) | .785 | [.718, .837] | >.80 | Threshold not reached |
| Parent-report | Weighted outdegree: ICC(A,1) | .732 | [.684, .773] | >.80 | Threshold not reached |

**Table S4a. Concurrent associations of PECAN characteristics with baseline PSC-D psychopathology.**

| **Informant** | **PECAN characteristic** | **Pearson r** | **95% CI** | **p** | **Spearman ρ** | **p** |
| --- | --- | --- | --- | --- | --- | --- |
| Self-report | Summed PECAN severity | .589 | [.503, .664] | <.001 | .475 | <.001 |
| Parent-report | Summed PECAN severity | .262 | [.075, .431] | .007 | .149 | .127 |
| Self-report | Mean problem severity / intensity | −.121 | [−.240, .002] | .054 | −.303 | <.001 |
| Parent-report | Mean problem severity / intensity | −.167 | [−.346, .025] | .088 | −.193 | .048 |
| Self-report | Mean perceived causal strength | −.186 | [−.302, −.065] | .003 | −.369 | <.001 |
| Parent-report | Mean perceived causal strength | −.257 | [−.427, −.070] | .008 | −.354 | <.001 |
| Self-report | Network density | −.211 | [−.325, −.090] | <.001 | −.358 | <.001 |
| Parent-report | Network density | −.088 | [−.274, .105] | .370 | −.179 | .066 |

**Note.** Pearson correlations were the primary linear-association estimates; Spearman correlations were rank-based sensitivities. Summed severity combines problem breadth and intensity, whereas mean severity is breadth-independent.

**Table S4b. Joint standardized regressions of perceived causal strength and density on baseline PSC-D.**

| **Informant** | **Predictor** | **Standardized β** | **95% CI** | **p** | **Model R²** |
| --- | --- | --- | --- | --- | --- |
| Self-report | Mean perceived causal strength | −.144 | [−.267, −.020] | .023 | .064 |
| Self-report | Network density | −.176 | [−.300, −.053] | .005 | .064 |
| Parent-report | Mean perceived causal strength | −.249 | [−.440, −.059] | .011 | .069 |
| Parent-report | Network density | −.050 | [−.241, .141] | .602 | .069 |

**Note.** Each informant-specific model included both relational characteristics simultaneously.

**Table S5. Preregistered T1–T4 trajectory analyses.**

| **Informant** | **Interaction** | **b** | **95% CI** | **p** | **Interpretation** |
| --- | --- | --- | --- | --- | --- |
| Self-report | Mean perceived causal strength × time | −.023 | [−.240, .194] | .835 | No evidence of differential symptom slope |
| Self-report | Network density × time | −.368 | [−.689, −.047] | .025 | Inverse association, opposite to preregistered direction; not robust across sensitivity specifications |
| Parent-report | Mean perceived causal strength × time | −.148 | [−.468, .173] | .367 | No evidence of differential symptom slope |
| Parent-report | Network density × time | −.319 | [−.689, .050] | .091 | No statistically reliable interaction |

**Note.** These preregistered models test whether baseline PECAN characteristics were associated with differential change across T1–T4. The main manuscript emphasizes baseline-adjusted T2–T4 models because they more directly estimate incremental prognostic information.

**Table S6a. Sensitivity analyses for the central problem-breadth association.**

| **Sensitivity** | **Informant** | **n participants** | **n follow-up obs.** | **Problem-breadth b** | **95% CI** | **p** | **ΔAIC** | **ΔBIC** | **LRT p** |
| --- | --- | --- | --- | --- | --- | --- | --- | --- | --- |
| PSC-D item breadth adjustment | Self-report | 161 | 399 | 1.219 | [.664, 1.774] | <.001 | −15.29 | −11.30 | <.001 |
| PSC-D item breadth adjustment | Parent-report | 74 | 183 | 1.528 | [.831, 2.224] | <.001 | −14.22 | −11.01 | <.001 |
| Source-subsample adjustment | Self-report | 161 | 399 | 1.253 | [.655, 1.852] | <.001 | — | — | — |
| Source-subsample adjustment | Parent-report | 74 | 183 | 1.474 | [.769, 2.178] | <.001 | — | — | — |
| Source × follow-up-time sensitivity | Self-report | 161 | 399 | 1.268 | [.673, 1.864] | <.001 | — | — | — |
| Source × follow-up-time sensitivity | Parent-report | 74 | 183 | 1.503 | [.792, 2.213] | <.001 | — | — | — |
| RSI > 2 excluded | Self-report | 157 | 389 | 1.281 | [.691, 1.871] | <.001 | — | — | — |
| RSI > 2 excluded | Parent-report | 60 | 141 | 1.275 | [.408, 2.141] | .004 | — | — | — |
| Age restricted to 14–17 years | Self-report | 22 | 37 | 1.515 | [.033, 2.997] | .045 | — | — | — |

Note. The PSC-D item-breadth model additionally adjusted for the number of baseline PSC-D items endorsed at least sometimes. Source robustness added source subsample as a fixed effect; the stricter source sensitivity additionally allowed the follow-up time trend to vary by source. The RSI sensitivity excluded participants with Response Speed Index > 2. In the age-restricted model, 22 participants contributed 37 available follow-up observations across T2–T4.

**Table S6b. Relationship between PECAN problem breadth and conventional PSC-D item breadth.**

| **Informant** | **n** | **PECAN breadth vs PSC-D item breadth: Pearson r** | **p** | **Spearman ρ** | **p** | **PSC-D item breadth vs PSC-D total: Pearson r** |
| --- | --- | --- | --- | --- | --- | --- |
| Self-report | 255 | .606 | <.001 | .637 | <.001 | .941 |
| Parent-report | 106 | .389 | <.001 | .360 | <.001 | .932 |

**Note.** PSC-D item breadth is an analytic comparator, not a validated PSC-D scale. It is the count of baseline items scored > 0 (at least sometimes), range 0–17.

**Table S6c. Relational estimates in selected robustness analyses.**

| **Sensitivity** | **Informant** | **Relational characteristic** | **b** | **95% CI** | **p** |
| --- | --- | --- | --- | --- | --- |
| Source-subsample adjustment | Self-report | Network density | −.611 | [−1.104, −.119] | .015 |
| Source × follow-up-time sensitivity | Self-report | Network density | −.614 | [−1.103, −.124] | .014 |
| RSI > 2 excluded | Self-report | Mean perceived causal strength | .418 | [−.010, .846] | .056 |
| RSI > 2 excluded | Self-report | Network density | −.584 | [−1.073, −.095] | .019 |
| RSI > 2 excluded | Self-report | Maximum normalized weighted outdegree | −.246 | [−.671, .180] | .257 |
| RSI > 2 excluded | Parent-report | Mean perceived causal strength | −.485 | [−1.268, .298] | .225 |
| RSI > 2 excluded | Parent-report | Network density | −.401 | [−1.286, .484] | .375 |
| RSI > 2 excluded | Parent-report | Maximum normalized weighted outdegree | −.360 | [−1.237, .517] | .421 |
| Age restricted to 14–17 years | Self-report | Mean perceived causal strength | .482 | [−.283, 1.247] | .217 |
| Age restricted to 14–17 years | Self-report | Network density | .335 | [−.708, 1.377] | .529 |
| Age restricted to 14–17 years | Self-report | Maximum normalized weighted outdegree | .407 | [−.240, 1.055] | .218 |

**Note.** Relational models are adjusted for baseline PSC-D, follow-up time, and problem breadth. The age-restricted density and outdegree estimates changed sign relative to the full self-report sample, reinforcing the limited robustness of relational findings.

**Table S7. Follow-up availability and selected baseline correlates of attrition.**

| **Informant** | **Availability outcome** | **Baseline predictor (standardized)** | **OR** | **95% CI** | **p** |
| --- | --- | --- | --- | --- | --- |
| Parent-report | At least one T2–T4 follow-up | Baseline PSC-D | 1.09 | [.64, 1.84] | .761 |
| Parent-report | At least one T2–T4 follow-up | Problem breadth | .47 | [.28, .80] | .006 |
| Parent-report | At least one T2–T4 follow-up | Network density | .86 | [.54, 1.36] | .513 |
| Parent-report | At least one T2–T4 follow-up | Mean perceived causal strength | 1.64 | [1.00, 2.70] | .052 |
| Parent-report | At least one T2–T4 follow-up | Age | 3.22 | [1.45, 7.16] | .004 |
| Parent-report | At least one T2–T4 follow-up | Source: parent_main vs parent_code | 4.87 | [.80, 29.70] | .086 |
| Parent-report | T4 available | Baseline PSC-D | 1.42 | [.85, 2.39] | .183 |
| Parent-report | T4 available | Problem breadth | .33 | [.19, .57] | <.001 |
| Parent-report | T4 available | Network density | .86 | [.55, 1.36] | .531 |
| Parent-report | T4 available | Mean perceived causal strength | 2.14 | [1.31, 3.50] | .002 |
| Parent-report | T4 available | Age | 1.47 | [.72, 3.01] | .286 |
| Parent-report | T4 available | Source: parent_main vs parent_code | 1.64 | [.32, 8.42] | .554 |
| Self-report | At least one T2–T4 follow-up | Baseline PSC-D | .47 | [.30, .74] | .001 |
| Self-report | At least one T2–T4 follow-up | Problem breadth | .70 | [.45, 1.11] | .131 |
| Self-report | At least one T2–T4 follow-up | Network density | .95 | [.68, 1.33] | .766 |
| Self-report | At least one T2–T4 follow-up | Mean perceived causal strength | 1.75 | [1.21, 2.54] | .003 |
| Self-report | At least one T2–T4 follow-up | Age | .43 | [.25, .74] | .002 |
| Self-report | At least one T2–T4 follow-up | Source: self_ju_code vs self_heranwachsende | .04 | [.01, .16] | <.001 |
| Self-report | At least one T2–T4 follow-up | Source: self_main vs self_heranwachsende | 1.55 | [.60, 4.02] | .369 |
| Self-report | T4 available | Baseline PSC-D | .52 | [.30, .90] | .020 |
| Self-report | T4 available | Problem breadth | .43 | [.24, .77] | .004 |
| Self-report | T4 available | Network density | .62 | [.39, .98] | .042 |
| Self-report | T4 available | Mean perceived causal strength | 4.10 | [2.41, 6.98] | <.001 |
| Self-report | T4 available | Age | .56 | [.29, 1.07] | .080 |
| Self-report | T4 available | Source: self_ju_code vs self_heranwachsende | .04 | [.01, .25] | <.001 |
| Self-report | T4 available | Source: self_main vs self_heranwachsende | 1.30 | [.43, 3.94] | .646 |

Note. Continuous predictors were standardized within informant group. Source coefficients are relative to the reference source shown in the predictor label. These analyses characterize observed baseline correlates of follow-up availability but cannot establish missing at random or rule out informative missingness.

**Table S8. Exploratory PSC-D internalizing and externalizing prognostic models.**

| **Status** | **Outcome** | **Informant** | **Predictor** | **b** | **95% CI** | **p** |
| --- | --- | --- | --- | --- | --- | --- |
| Exploratory secondary outcome | PSC Internalizing | Self-report | Problem breadth | 1.022 | [.740, 1.305] | <.001 |
| Exploratory secondary outcome | PSC Internalizing | Parent-report | Problem breadth | .549 | [.216, .881] | .001 |
| Exploratory secondary outcome | PSC Externalizing | Self-report | Problem breadth | .430 | [.191, .669] | <.001 |
| Exploratory secondary outcome | PSC Externalizing | Parent-report | Problem breadth | .721 | [.355, 1.088] | <.001 |
| Exploratory secondary outcome | PSC Internalizing | Self-report | Mean perceived causal strength | .130 | [−.100, .360] | .268 |
| Exploratory secondary outcome | PSC Internalizing | Self-report | Network density | −.075 | [−.345, .194] | .584 |
| Exploratory secondary outcome | PSC Internalizing | Self-report | Maximum normalized weighted outdegree | .009 | [−.223, .242] | .936 |
| Exploratory secondary outcome | PSC Internalizing | Parent-report | Mean perceived causal strength | −.112 | [−.430, .206] | .491 |
| Exploratory secondary outcome | PSC Internalizing | Parent-report | Network density | −.273 | [−.609, .062] | .111 |
| Exploratory secondary outcome | PSC Internalizing | Parent-report | Maximum normalized weighted outdegree | −.226 | [−.575, .123] | .205 |
| Exploratory secondary outcome | PSC Externalizing | Self-report | Mean perceived causal strength | .010 | [−.225, .245] | .931 |
| Exploratory secondary outcome | PSC Externalizing | Self-report | Network density | −.224 | [−.491, .042] | .099 |
| Exploratory secondary outcome | PSC Externalizing | Self-report | Maximum normalized weighted outdegree | −.159 | [−.383, .065] | .164 |
| Exploratory secondary outcome | PSC Externalizing | Parent-report | Mean perceived causal strength | −.332 | [−.714, .050] | .089 |
| Exploratory secondary outcome | PSC Externalizing | Parent-report | Network density | −.131 | [−.552, .291] | .544 |
| Exploratory secondary outcome | PSC Externalizing | Parent-report | Maximum normalized weighted outdegree | −.028 | [−.465, .409] | .899 |

Note. Problem-breadth models adjusted for the corresponding baseline PSC-D subscale and follow-up time. Relational models additionally adjusted for problem breadth. Internalizing and externalizing outcomes were exploratory secondary analyses and do not alter the interpretation of the PSC-D total-score analyses.

**Appendix S1.** STROBE Checklist

| **Section** | **Item** | **Recommendation** | **Page No.** |
| --- | --- | --- | --- |
| Title and abstract | 1(a) | Indicate the study’s design with a commonly used term in the title or abstract | **2** |
|  | 1(b) | Provide an informative and balanced summary of what was done and what was found | **2** |
| Introduction – Background/rationale | 2 | Explain the scientific background and rationale | **3–6** |
| Objectives | 3 | State specific objectives, including prespecified hypotheses | **6** |
| Methods – Study design | 4 | Present key elements of study design early in the paper | **7** |
| Setting | 5 | Describe setting, locations, dates, recruitment and follow-up | **7** |
| Participants | 6(a) | Give eligibility criteria, sources and methods of selection and follow-up | **7; Figure S1** |
| Participants | 6(b) | Matching criteria, if applicable | **N/A** |
| Variables | 7 | Clearly define outcomes, exposures, predictors, confounders, effect modifiers | **8–13** |
| Data sources / measurement | 8 | Give sources of data and details of assessment methods | **8–10** |
| Bias | 9 | Describe efforts to address potential sources of bias | **7, 12–13, 19–20** |
| Study size | 10 | Explain how the study size was arrived at | **7** |
| Quantitative variables | 11 | Explain how quantitative variables were handled | **9–13** |
| Statistical methods | 12(a) | Describe all statistical methods, including confounding control | **10–13** |
|  | 12(b) | Methods for subgroups/interactions | **12–13** |
|  | 12(c) | Explain how missing data were addressed | **11, 14** |
|  | 12(d) | Explain how loss to follow-up was addressed | **12–14; Table S7** |
|  | 12(e) | Describe sensitivity analyses | **12–13** |
| Results – Participants | 13(a) | Report numbers at each stage | **7, 13–14; Figure S1** |
|  | 13(b) | Give reasons for non-participation | **7; Figure S1** |
|  | 13(c) | Consider a flow diagram | **Fig. S1** |
| Descriptive data | 14(a) | Give participant characteristics | **13; Table 1** |
|  | 14(b) | Indicate missing data | **13–14; Table S7** |
|  | 14(c) | Summarize follow-up time | **7, 13** |
| Outcome data | 15 | Report outcome events or summary measures | **13–16** |
| Main results | 16(a) | Give unadjusted and adjusted estimates with precision | **15–16; Figure 3** |
|  | 16(b) | Report category boundaries when continuous variables were categorized | **8–13** |
|  | 16(c) | Translate relative risk into absolute risk where relevant | **N/A** |
| Other analyses | 17 | Report subgroup/sensitivity analyses | **16; Tables S5-S8** |
| Discussion – Key results | 18 | Summarize key results | **17** |
| Limitations | 19 | Discuss limitations and direction/magnitude of bias | **19–20** |
| Interpretation | 20 | Give cautious overall interpretation | **17–21** |
| Generalisability | 21 | Discuss external validity | **19–21** |
| Other information – Funding | 22 | State funding and role of funders | **21** |
